# Effects of genetic polymorphisms on the OCT1 and OCT2-mediated uptake of ranitidine

**DOI:** 10.1371/journal.pone.0189521

**Published:** 2017-12-13

**Authors:** Marleen Julia Meyer, Tina Seitz, Jürgen Brockmöller, Mladen Vassilev Tzvetkov

**Affiliations:** Institute of Clinical Pharmacology, University Medical Center Göttingen, Göttingen, Germany; University of Kentucky, UNITED STATES

## Abstract

**Background:**

Ranitidine (Zantac^®^) is a H_2_-receptor antagonist commonly used for the treatment of acid-related gastrointestinal diseases. Ranitidine was reported to be a substrate of the organic cation transporters OCT1 and OCT2. The hepatic transporter OCT1 is highly genetically variable. Twelve major alleles confer partial or complete loss of OCT1 activity. The effects of these polymorphisms are highly substrate-specific and therefore difficult to predict. The renal transporter OCT2 has a common polymorphism, Ala270Ser, which was reported to affect OCT2 activity.

**Aim:**

In this study we analyzed the effects of genetic polymorphisms in *OCT1* and *OCT2* on the uptake of ranitidine and on its potency to inhibit uptake of other drugs.

**Methods and results:**

We characterized ranitidine uptake using HEK293 and CHO cells stably transfected to overexpress wild type OCT1, OCT2, or their naturally occurring allelic variants. Ranitidine was transported by wild-type OCT1 with a K_m_ of 62.9 μM and a v_max_ of 1125 pmol/min/mg protein. Alleles OCT1*5, *6, *12, and *13 completely lacked ranitidine uptake. Alleles OCT1*2, *3, *4, and *10 had v_max_ values decreased by more than 50%. In contrast, OCT1*8 showed an increase of v_max_ by 25%. The effects of *OCT1* alleles on ranitidine uptake strongly correlated with the effects on morphine uptake suggesting common interaction mechanisms of both drugs with OCT1. Ranitidine inhibited the OCT1-mediated uptake of metformin and morphine at clinically relevant concentrations. The inhibitory potency for morphine uptake was affected by the OCT1*2 allele. OCT2 showed only a limited uptake of ranitidine that was not significantly affected by the Ala270Ser polymorphism.

**Conclusions:**

We confirmed ranitidine as an OCT1 substrate and demonstrated that common genetic polymorphisms in *OCT1* strongly affect ranitidine uptake and modulate ranitidine’s potential to cause drug-drug interactions. The effects of the frequent *OCT1* polymorphisms on ranitidine pharmacokinetics in humans remain to be analyzed.

## Introduction

Ranitidine (Zantac^®^) is a histamine H_2_-receptor antagonist which is used for the treatment of acid-related gastrointestinal diseases such as pyrosis (heartburn) and gastric ulcers. Ranitidine is still broadly used. Along with omeprazole, ranitidine is listed by the World Health Organization (WHO) as an essential anti-ulcer agent [1].

While proton pump inhibitors (PPIs) have mostly superseded H_2_-antagonists like ranitidine, there are some reserves and contraindications against PPIs making ranitidine a drug of choice in many people including elderly people more susceptible to clostridium infections [2, 3]. Furthermore, recent pharmacovigilance analyses suggested a higher risk of death in individuals using PPIs, compared to individuals using H_2_-antagonists including ranitidine [4].

Ranitidine is sold over the counter in many countries. Some adverse reactions were reported in ranitidine users, including headache and upper respiratory tract infections [5]. However, meta-analyses of controlled clinical trials failed to show a direct relation of any adverse effects with ranitidine administration [6]. On the other hand, ranitidine administration was related to rare idiosyncratic liver toxicity [7].

Ranitidine is a hydrophilic, weakly basic compound. At physiological pH of 7.4 86% of ranitidine molecules are positively charged organic cations. Maximal plasma concentrations of ranitidine are reached two to three hours after administration with an oral bioavailability of 50–60% [8]. After oral administration approximately half of ranitidine is eliminated unchanged via renal excretion. The remaining up to 50% is metabolized in the liver, the N and S-oxides via flavin-containing monooxygenases (FMOs), and the demethylated metabolite via cytochrome P450 enzymes. Biliary excretion does not play an important role [8, 9]. Hepatic dysfunction leads to an increase in bioavailability from 50 to 70% [10]. Little is known about the specific mechanisms and transporters involved in ranitidine absorption and elimination. However, ranitidine was suggested to be a substrate of the human organic cation transporters 1 (OCT1) and 2 (OCT2) [11].

OCT1 (alternative name *SLC22A1*) is a polyspecific transporter that is strongly expressed in the sinusoidal membranes of human hepatocytes. OCT1 mediates the uptake of cationic and weakly basic substances into the liver. Known OCT1 substrates are numerous drugs, toxins, and some endogenous substances like thiamine (vitamin B1) [12–21].

OCT2 (alternative name *SLC22A2*) is strongly expressed in the basolateral membranes of epithelial cells in kidney proximal tubules [22, 23]. OCT2 mediates the uptake of organic cations from the blood into the renal tubule and thereby is involved in the first step of renal excretion of organic cations [24]. Known OCT2 substrates are toxins, drugs like metformin and cisplatin, and endogenous compounds such as creatinine and tryptophan [23, 25–29].

The *OCT1* gene is highly polymorphic in humans. In Europeans and White Americans, slow and deficient OCT1 transport is mostly explained by five alleles: OCT1*2 (characterized by a deletion of Met420), *3 (Arg61Cys), *4 (Gly401Ser), *5 (Gly465Arg/Met420del), and *6 (Cys88Arg/Met420del) [14, 30]. Nine percent of Europeans and White Americans are homozygous or compound heterozygous carriers of these loss-of-function alleles (so called poor OCT1 transporters) [14, 16, 30]. An additional 40% of Europeans and White Americans are heterozygous carriers of these alleles and have only one active copy of *OCT1* in their genomes. Poor OCT1 transporters were reported to have altered pharmacokinetics and efficacy of the drugs metformin, morphine, tropisetron, tramadol, bendamustine, sumatriptan, and fenoterol [12, 16, 17, 31–35, 36, Tzvetkov, 2017 #350]. The reports of the effects on morphine, however, are not univocal [37] (for review see [38]). Polymorphisms and tumor-specific somatic mutations in OCT1 have also been suggested to confer reduced sensitivity to sorafenib [39]. The number of poor OCT1 transporters varies strongly among different ethnicities and different world regions [14, 19]. While rare in East Asia, poor OCT1 transporters represent more than 80% of certain populations in South America (e.g. the Surui Indians) [19].

A number of *OCT1* alleles, i.e. OCT1*2, *7, *10, *11, and *13, show a substrate-specific loss of activity [14, 15, 19, 34]. OCT1*2, the most common variant *OCT1* allele (global allele frequency of 12.2%), shows strong substrate-specific effects. While there was no difference between OCT1*2 and wild type in the uptake of the model substrates MPP^+^ and ASP^+^ and the antimigraine drug sumatriptan [14, 19, 36], the OCT1*2 allele confers strongly reduced uptake of metformin, morphine, and thiamine [12, 15, 34], and complete loss of uptake of tropisetron and O-desmethyltramadol [16, 17]. The allele OCT1*10 (Ser189Leu) showed no difference in ASP^+^ uptake, a substantial reduction in metformin, thiamine, and tropisetron uptake, but an increase in morphine, debrisoquine, and monocrotaline uptake [19]. Due to the high substrate-specific effects drugs should be analyzed individually in order to predict the potential therapeutic consequences of the common *OCT1* alleles.

*OCT2* is less polymorphic than *OCT1* and particularly a complete lack of activity appears to be very rare. Ala270Ser is the only common functional polymorphism and leads to a moderate, substrate-dependent decrease in OCT2 transport activity [40–45]. The Ser270 allele was associated with 21% increased maximal uptake rates, but more than twice lower affinity to ipratropium [46]. The effects on metformin and the model substrate MPP^+^ are contradictory [47].

The aim of the present study was to investigate the effects of common genetic polymorphisms in *OCT1* and *OCT2* on the uptake of ranitidine. Furthermore, we wanted to investigate whether ranitidine may inhibit OCT1 at clinically relevant concentrations and whether the potency of this inhibition may be different depending on *OCT1* genetic variants. Such data may be an important prerequisite for further clinical investigation of the interindividual variation in ranitidine pharmacokinetics and therapeutic effects and may help evaluate the potential for using ranitidine as an *in vivo* inhibitor of OCT1.

## Materials and methods

### Cell lines and reagents

HEK293 or CHO cells overexpressing the human *OCT1* alleles *1A (characterized by the amino acid substitution Met408Val), *1B, *1C (Phe160Leu), *1D (Pro341Leu/Met408Val), *2 (Met420del), *3 (Arg61Cys), *4 (Gly401Ser), *5 (Gly465Arg/Met420del), *6 (Cys88Arg/Met420del), *7 (Ser14Phe), *8A (Arg488Met), *8B (Arg488Met/Met408Val), *9 (Pro117Leu), *10 (Ser189Leu), *11 (Ile449Thr), *12 (Ser29Leu), or *13 (Thr245Met), the human *OCT2* reference and variant alleles (Ala270Ser), human *OCT3*, and the control cells (transfected with the empty pcDNA5 vector) were generated by targeted chromosomal integration using the Flp-In™ System (Life Technologies, Darmstadt, Germany). The generation and characterization of the cells was described in detail before [19, 46, 48].

Ranitidine, 1-methyl-4-phenylpyridinium (MPP^+^), codeine, morphine, and metformin were obtained from Sigma Aldrich (Taufkirchen, Germany). Deuterium-labeled ranitidine (ranitidine-d6) was obtained from Toronto Research Chemicals (TRC, Toronto, ON, Canada), buformin from Wako Chemicals (Neuss, Germany), and 4-(4-(dimethylamino)styryl)-N-methylpyridinium (ASP^+^) was obtained from Life Technologies (Darmstadt, Germany). Tritium-labeled MPP^+^ (N-[methyl-^3^H], 80 Ci/mmol) was obtained from Hartmann Analytic (Braunschweig, Germany).

Dulbecco’s Modified Eagle Medium (DMEM), Roswell Park Memorial Institute (RPMI) 1640, Hank’s Buffered Salt Solution (HBSS), and additives used for cell culturing were obtained from Life Technologies. Poly-D-lysine (1–4 kDa), 2-[4-(2-hydroxyethyl)piperazin-1-yl]ethanesulfonic acid (HEPES), bicinchoninic acid, and copper sulfate pentahydrate were obtained from Sigma Aldrich. Twelve-well plates were obtained from Nunc (Langenselbold, Germany). Acetonitrile and methanol in LC-MS/MS grade were obtained from LGC Standards (Wesel, Germany), formic acid (LC-MS/MS grade), and sodium chloride were obtained from Merck (Darmstadt, Germany). Sodium dodecylsulfate (SDS, ultrapure) was obtained from AppliChem (Darmstadt, Germany). Aquasafe Plus liquid scintillator and 20 ml polyvials were purchased from Zinsser Analytics (Frankfurt am Main, Germany).

### Cellular uptake measurements

Six hundred thousand HEK293 or CHO cells were plated per well in 12-well plates (pre-coated with poly-D-lysine) and were grown for 48 hours to reach confluence. Cells were kept at 37°C and 5% CO_2_ in DMEM (HEK293) or RPMI (CHO), supplemented with 10% FCS, 100 U/ml penicillin, and 100 μg/ml streptomycin. Uptake experiments were performed at pH 7.4 using HBSS supplemented with 10 mM HEPES buffer (herein referred to as HBSS+). Cells were washed once with 1 ml pre-warmed HBSS+ buffer (37°C). The reaction was started by adding 400 μl pre-warmed HBSS+ containing varying concentrations of ranitidine in the presence or absence of 1 mM MPP^+^ as inhibitor and stopped by adding 2 ml ice-cold HBSS+. For concentration-dependent measurements, ranitidine was used in concentrations between 10 and 600 μM and uptake was allowed for 2 min. For time-dependent measurements, 1 μM ranitidine was used and the reaction was stopped after 1, 2, 3, 5, 10, or 15 min. For inhibition experiments, the uptake of 1 μM ASP^+^, 0.1 μM MPP^+^, 100 μM metformin, or 0.1 μM morphine was measured for 2 min with ranitidine as inhibitor in concentrations between 0.1 and 5000 μM. Cells were washed twice with 2 ml ice-cold HBSS+ and lysed in 500 μl lysis buffer. Depending on the detection method, cells were lysed in RIPA buffer for fluorescence spectroscopy (ASP^+^), in 80% acetonitrile containing internal standards for LC-MS/MS detection (10 ng/ml ranitidine-d6 for ranitidine, 50 ng/ml buformin for metformin, and 10 ng/ml codeine for morphine measurement), or in 0.1 M NaOH/0.1% SDS for scintillation counting (MPP^+^).

The intracellular concentration of MPP^+^ was determined using liquid scintillation counting of radioactively labeled substrate. To this end, 400 μl cell lysate was measured with 9 ml Aquasafe Plus liquid scintillator using the Scintillation Counter LS6500 (Beckman Coulter, Krefeld, Germany). Intracellular ASP^+^ concentrations were determined using fluorescence spectroscopy. To this end, 200 μl cell lysate was measured in a Tecan Ultra Microplate Reader (Tecan Group AG, Männedorf, Switzerland) at wave lengths of 485 nm (excitation) and 612 nm (emission). Intracellular concentrations of ranitidine, metformin, and morphine were determined using LC-MS/MS as described below.

For all transport measurements, the intracellular amount of substrate was normalized to the total amount of protein of the sample as measured using the bicinchoninic acid assay [49].

### Quantification of ranitidine, metformin, and morphine by LC-MS/MS

For quantification of intracellular substrates, the cell debris were removed by centrifugation at 17,000 x g for 10 min. Four-hundred μl of the supernatant was evaporated to dryness under nitrogen flow at 40°C. The residue was reconstituted in 200 μl 0.1% formic acid and 10 μl were injected into the LC-MS/MS. For the LC-MS/MS analysis an API 4000^™^ system tandem mass spectrometer (AB SCIEX, Darmstadt, Germany) was used. Samples were separated using a Brownlee SPP RP-Amide Column (4.6x100 mm, 2.7 μm; PerkinElmer), with a SecurityGuard C18, 4x2 mm pre-column (Phenomenex, Aschaffenburg, Germany). Elution was achieved with a mobile phase of 0.1% (v/v) formic acid and varying concentrations of organic solvent (Table 1) at a flow rate of 300 μl/min. Substrates were detected using the multiple reaction monitoring (MRM) mode of transition using the parameters listed in Table 1. The detection was linear within the range of 1 nM to 2.5 μM for ranitidine, 3 nM to 300 nM for metformin, and 0.2 nM to 10 nM for morphine with coefficients of variation below 7%, 11.5%, and 9% for ranitidine, metformin, and morphine, respectively.

**Table 1:** LC-MS/MS parameters for the detection of intracellular substrate concentration.

Analyte	Mass transition [m/z]	Retention time [min]	Internal standard (IS)	Mass transition of IS [m/z]	Retention time of IS [min]	Organic solvent in mobile phase [%]*
Ranitidine	315.3>130.1	4.2	Ranitidine-d6	321.2>130.1	4.2	8
Metformin	130.0>71.0	2.78	Buformin	128>60.0	4.04	3
Morphine	286.2>201.1	3.77	Codeine	300.3>215.1	5.2	8

* The organic solvent consists of 6 volume parts acetonitrile and one part methanol.

### Data analyses

Nonlinear regression to the Michaelis-Menten equation was performed to determine K_m_ and v_max_ using GraphPad Prism version 6 (GraphPad Software Inc., La Jolla, CA, USA). Nonlinear regression to the Hill equation

$$v\;=\;v_{min}+\frac{\left(v_{max}-v_{min}\right)}{{1+(\frac{\left[I\right]}{{IC}_{50}})}^{-Hillslope}}$$

was performed to determine IC_50_ using SigmaPlot version 12.0 (Systat Software, San Jose, California, USA).

The effects of genetic variants on the cellular uptake of ranitidine (K_m_ and v_max_) and on ranitidine-mediated inhibition (IC_50_) were compared using analyses of variance (ANOVA) followed by Tukey’s Honestly Significant Difference (HSD) post hoc analyses. SPSS version 23 (SPSS Inc., IBM, Chicago, IL, USA) was used for statistical analyses.

## Results

### Ranitidine as a substrate of OCT1

**Fig 1:**
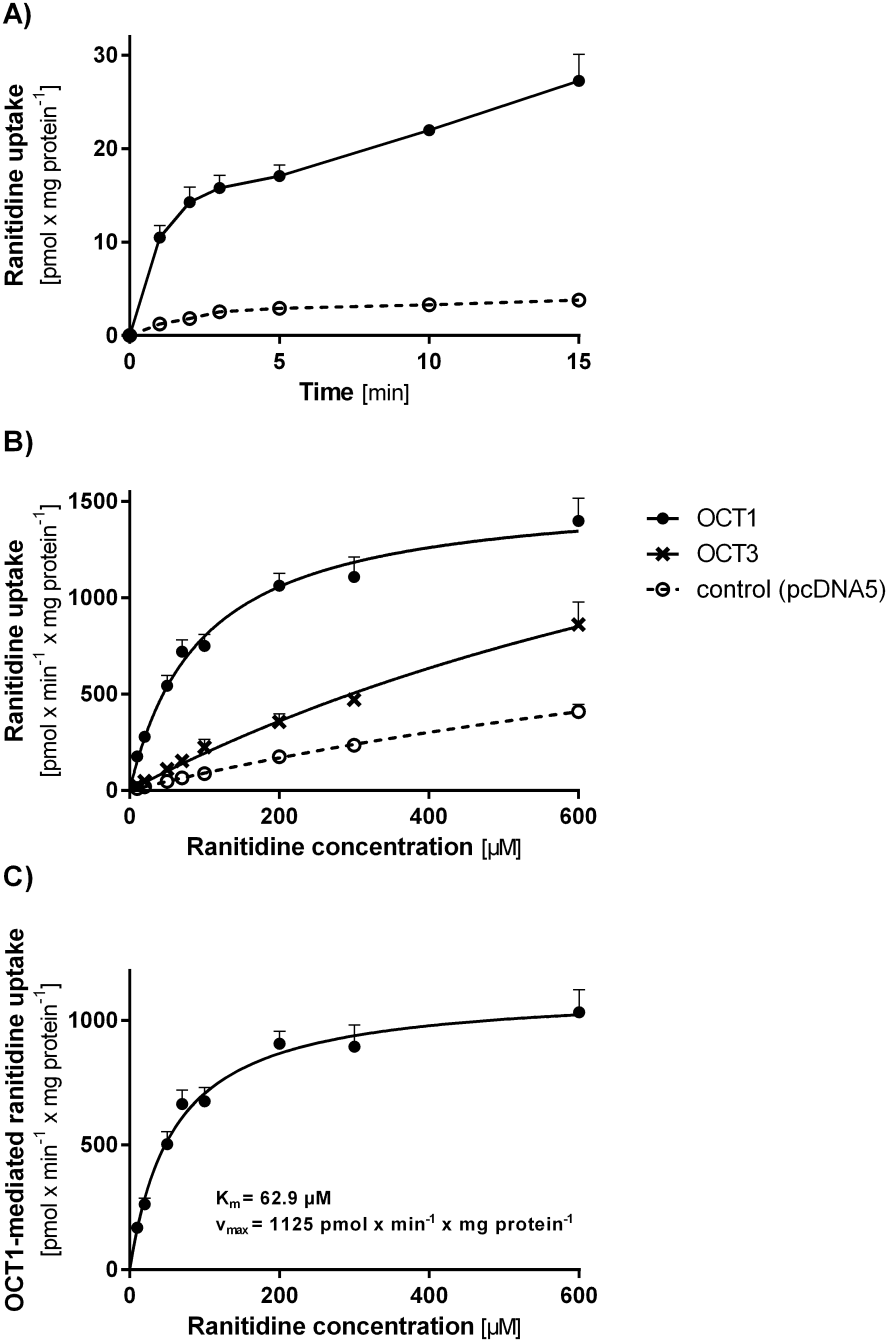
Ranitidine uptake via OCT1 and OCT3. A) Time-dependence of ranitidine uptake via OCT1. HEK293 T-REx™ cells stably transfected to overexpress OCT1 (filled circles) and control cells transfected with the empty vector pcDNA5 (open circles) were incubated for up to 15 min with 1 μM ranitidine. The data is shown as means and standard error of the means of at least three independent experiments. B) and C) Concentration-dependence of ranitidine uptake via OCT1 and OCT3. OCT1 and OCT3 (crosses)-overexpressing and control cells were incubated for 2 min with increasing concentrations of ranitidine (ranging from 10 to 600 μM). The OCT1-mediated portion of ranitidine uptake (C) was calculated using the data from panel B by subtracting the uptake of control cells from the uptake of OCT1-overexpressing cells. The data is shown as means and standard error of the means of at least three independent experiments.

To evaluate ranitidine as a substrate of OCT1 we compared the intracellular accumulation of ranitidine between HEK293 cells overexpressing wild type OCT1 and control cells transfected with the empty vector pcDNA5. OCT1-overexpressing cells showed both a time and concentration-dependent increase in the intracellular concentration of ranitidine (Fig 1A and 1B). Compared to the control cells, the intracellular concentration of ranitidine in the OCT1-overexpressing cells was 8.5-fold higher after 1 min of incubation.

In concentration-dependent experiments a mixture of saturable and unsaturable uptake was observed for the OCT1-overexpressing cells (Fig 1B). To determine the OCT1-mediated portion of the ranitidine uptake, the uptake of the control cells was subtracted from the uptake of the OCT1-overexpressing cells. The OCT1-related ranitidine uptake showed Michaelis-Menten kinetics with a K_m_ of 62.9 ± 4.32 μM and v_max_ of 1125 ± 86.1 pmol x min^-1^ x mg protein^-1^ (Fig 1C). The K_m_ value in our study is very similar to the literature data (70 ± 9 μM, [11]).

We further analyzed whether OCT3, the other organic cation transporter expressed in the sinusoidal membrane of the human liver, also transports ranitidine. HEK293 cells stably overexpressing OCT3 showed only up to 2-fold increase in ranitidine uptake compared to the controls (Fig 1B). At clinically relevant concentrations the OCT3-mediated uptake was more than 14-fold lower than the OCT1-mediated uptake of ranitidine. It should also be considered that OCT3 expression in the human liver is more than 13-fold lower that OCT1 expression [12, 50]. Thus OCT1 is the major uptake transporter of ranitidine in the human liver.

### Effects of *OCT1* genetic polymorphisms on the cellular uptake of ranitidine

After confirming ranitidine as OCT1 substrate, we analyzed the effects of common *OCT1* polymorphisms on ranitidine uptake. We compared the uptake between the reference allele and the common *OCT1* variant alleles using two different concentrations of ranitidine, 1 and 10 μM. The concentrations were chosen to be representative for the maximal unbound plasma (C_max_) and the estimated maximal unbound portal vein concentration (C_max port_). The C_max_ of ranitidine varies between 0.7 and 1.7 μM after an oral administration of a single dose of 100 or 150 mg ranitidine [8]. Using the equation of Ito *et al*. [51, 52] the corresponding C_max port_ could be estimated to be between 20 and 30 μM.

**Fig 2:**
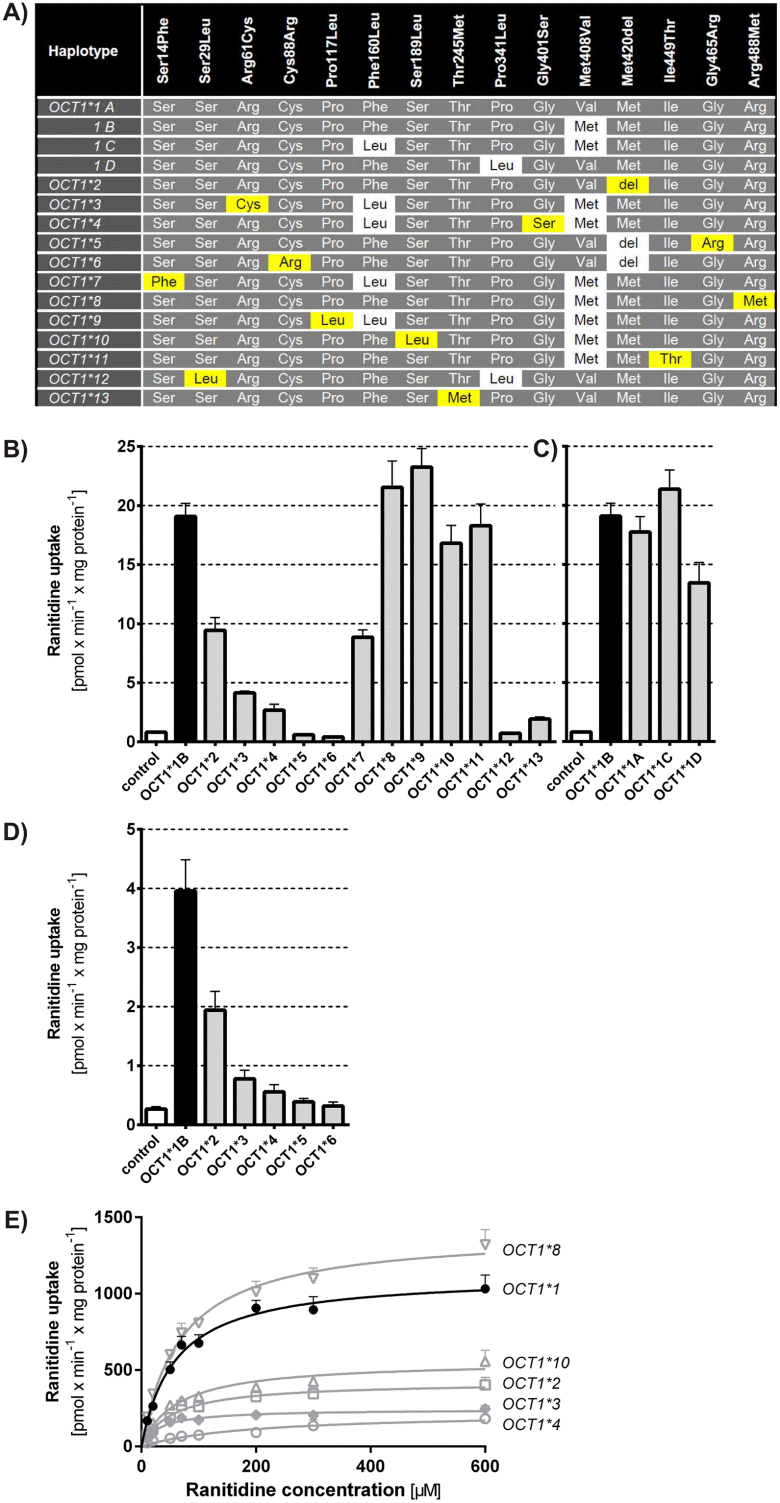
Effects of *OCT1* polymorphisms on ranitidine uptake. A) *OCT1* alleles analyzed in this study. Amino acid substitutions known to affect OCT1 function are highlighted in yellow. Amino acid substitutions known to not significantly affect OCT1 function are highlighted in white. B) to E) Effects of *OCT1* allelic variants on ranitidine uptake. HEK293 T-REx^™^ (B, C, E) and CHO (D) cells overexpressing the reference allele (OCT1*1B) and common *OCT1* allelic variants (the allelic variants are listed in panel A) were incubated with 1 μM (B to D) or with increasing concentrations (E) of ranitidine for 2 min. The OCT1-mediated uptake was calculated by subtracting the uptake of control cells from the uptake of OCT1-overexpressing cells. The data is shown as means and standard error of the means of at least three independent experiments.

We analyzed the major alleles OCT1*2 to *13 (Fig 2B), which have been previously described to affect OCT1 activity with other substrates [12, 13, 15–19, 33, 34, 48] and the sub-allelic variants OCT1*1B, *1C, and *1D (Fig 2C). The selected alleles represent 98.9% of the *OCT1* alleles currently known worldwide [19]. Two sub-alleles of allele OCT1*8, *8A and *8B, were analyzed, but showed no differences in ranitidine uptake, therefore the data was combined in the further analyses. The alleles OCT1*5, *6, *12, and *13 showed a complete lack of ranitidine transport activity after incubation with both 1 μM (Fig 2B) and 10 μM concentration of ranitidine (S1 Fig). Alleles OCT1*1A, *1C, *1D, *7, *9, and *11 showed no significant difference in the uptake of ranitidine compared to the reference allele (Fig 2B and 2C, S1 Fig). The effects of alleles OCT1*1 to *6 on ranitidine uptake were confirmed using an alternative cell model—stably transfected CHO cells (Fig 2D). The remaining *OCT1* variants were further analyzed by performing concentration-dependent uptake measurements. The alleles OCT1*2, *3, *4, and *10 showed a significant decrease of v_max_ (Fig 2E and Table 2). The decrease ranged from 50% (OCT1*10) to 91% (OCT1*4). These alleles also showed a significant decrease in the intrinsic clearance (CL_int_), ranging from 52% (OCT1*10) to 83% (OCT1*4). The OCT1*8 allele showed a 25% increase in v_max_, though the difference was not statistically significant (P = 0.5, Table 2, Fig 2E). In contrast, none of the analyzed polymorphisms significantly affected the affinity (K_m_) of ranitidine uptake (P = 0.17, Table 2).

**Table 2:** Effects of common amino acid substitutions on the kinetics of the OCT1-mediated uptake of ranitidine.

*OCT1* allele	Characteristic amino acid substitution	K_m_ [μM] (± SEM)	V_max_ [pmol x min^-1^ x mg protein^-1^] (±SEM)	CL_int_ [ml x min^-1^ x mg protein^-1^] (±SEM)
OCT1*1B	Reference allele	62.91 (±4.32)	1125.41 (±86.12)	18.48 (±1.83)
OCT1*1A	Met408Val	54.50 (±7.17)	1047.13 (±123.39)	19.32 (±0.95)
OCT1*1C	Phe160Leu	82.62 (±10.65)	1245.87 (±209.39)	15.05 (±1.74)
OCT1*1D	Pro341Leu/Met408Val	66.74 (±6.14)	883.10 (±76.48)	13.62 (±1.62)
OCT1*2	Met420del	52.25 (±8.38)	402.04 (±51.24)***	8.18 (±1.39)**
OCT1*3	Arg61Cys	39.45 (±7.35)	255.08 (±12.75)***	7.76 (±1.80)**
OCT1*4	Gly401Ser	56.40 (±28.25)	106.65 (±26.70)***	3.14 (±0.82)***
OCT1*7	Ser14Phe	61.58 (±13.19)	753.08 (±49.95)	14.94 (±3.61)
OCT1*8	Arg488Met	69.21 (±5.88)	1412.43 (±108.88)	20.68 (±1.22)
OCT1*9	Pro117Leu	77.17 (±33.23)	1138.47 (±279.72)	17.01 (±2.72)
OCT1*10	Ser189Leu	64.44 (±7.94)	567.50 (±59.10)*	8.957 (±0.75)*
OCT1*11	Ile449Thr	131.41 (±58.37)	1283.33 (±319.87)	11.56 (±2.19)

* P<0.05, ** P<0.01, *** P<0.001 compared to the reference allele in a Tukey’s HSD post hoc analysis following one-way ANOVA (P<10^−11^)

### Drug-drug-gene interactions: Effects of *OCT1* polymorphisms on the potency of ranitidine to inhibit OCT1 uptake

Ranitidine has been previously shown to inhibit the uptake of model OCT1 substrates such as MPP^+^ [11, 53], but the effect on clinically relevant drugs is still unknown. We analyzed the potency of ranitidine to inhibit the uptake of the drugs metformin and morphine as well as of the model substrates ASP^+^ and MPP^+^. More importantly, we analyzed whether the inhibitory potency of ranitidine is affected by the most common allelic variant OCT1*2 (Met420del). Ranitidine concentrations were chosen to cover the estimated ranitidine concentrations in plasma, portal vein, and gut after oral administration of 150 to 300 mg ranitidine.

**Fig 3:**
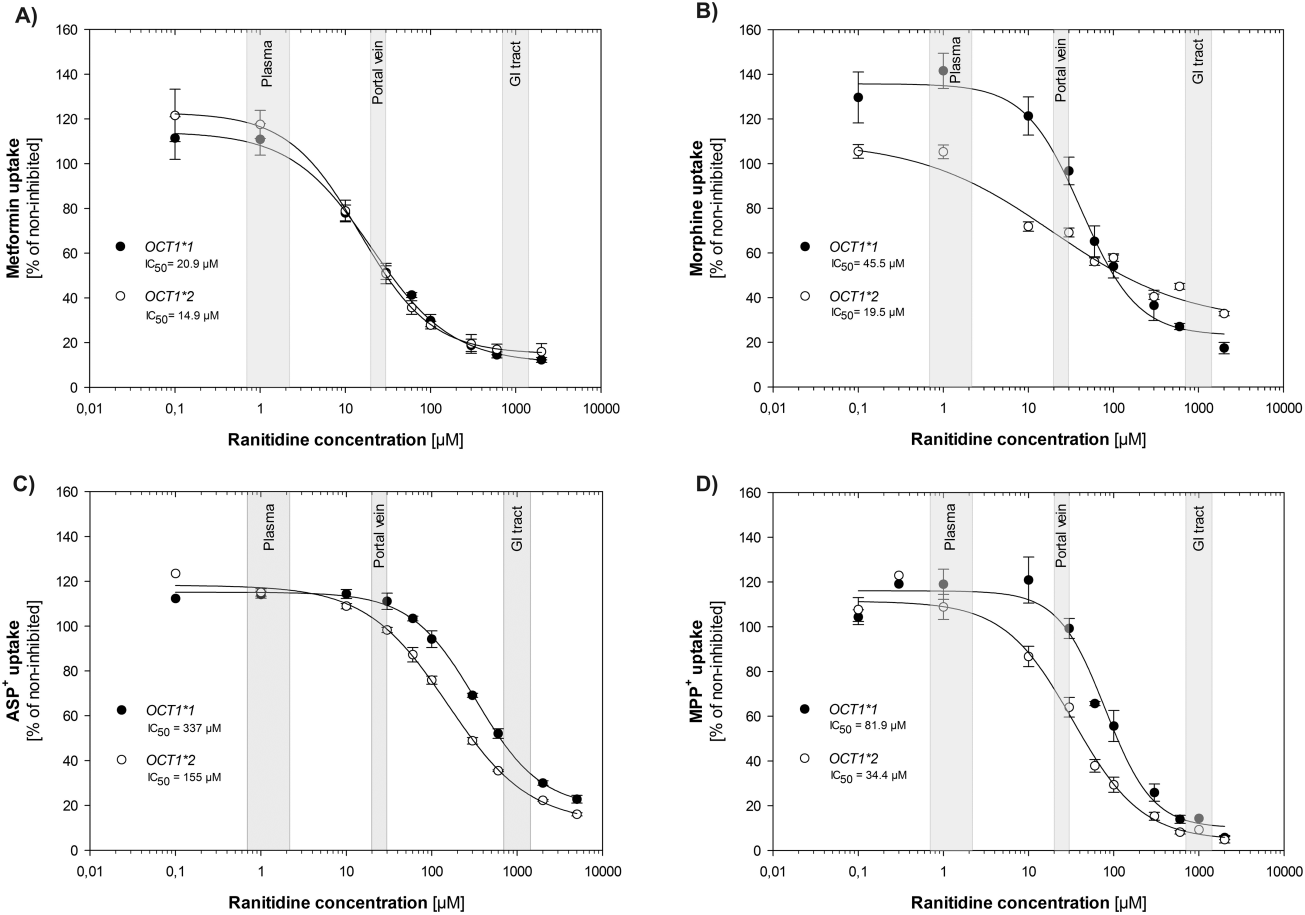
Genotype-dependence of the ranitidine inhibitory potency. We compared the potency of ranitidine to inhibit the uptake of metformin (A), morphine (B), ASP^+^ (C), and MPP^+^ (D) by the reference and OCT1*2 alleles. HEK293 T-REx™ cells overexpressing the reference or OCT1*2 allele were incubated for 2 min with 0.1 μM morphine (A), 100 μM metformin (B), 1 μM ASP^+^ (C), or 0.1 μM MPP^+^ (D) in the presence of increasing concentrations of ranitidine. The uptake is represented as percentage of OCT1-mediated ranitidine uptake without inhibition. Shown are means and standard error of the means of at least three independent experiments and the half maximal inhibitory concentrations (IC_50_) were calculated. The clinically relevant concentrations of ranitidine in plasma, portal vein, and gastrointestinal (GI) tract are highlighted in grey. The plasma concentrations were estimated based on the C_max_ of ranitidine after an oral administration of a single dose of 150 to 300 mg ranitidine [5, 8]. The concentrations in the portal vein were estimated as described elsewhere [51, 52]. The concentrations in the GI tract were estimated supposing that 150 to 300 mg ranitidine are solved in 686 ml GI fluid volume [54].

We observed highly substrate-dependent and moderately genotype-dependent differences in the potency of ranitidine to inhibit OCT1 uptake. Depending on the substrate, the inhibitory potency of ranitidine varied from an IC_50_ of 20.9 μM (metformin) to 337 μM (ASP^+^). Depending on the genotype, ranitidine was on average two-fold more potent in inhibiting the common OCT1*2 variant than the reference *OCT1* allele. The genotype-dependent differences were most prominent when inhibiting morphine (IC_50_ of 19.5 and 45.5 μM for OCT1*2 and *1, respectively) and least prominent when inhibiting metformin (IC_50_ of 14.8 and 20.9 μM for OCT1*2 and *1, respectively). At expected maximal plasma concentrations of ranitidine we did not observe any relevant inhibition of OCT1 uptake (Fig 3). Interestingly, ranitidine did not inhibit, but induced morphine uptake by 41% at C_max_. The induction was genotype-specific and was observed only in the reference, but not in the OCT1*2 allele (Fig 3B). At expected portal vein concentrations, we observed 50% inhibition of metformin uptake independent of the OCT1 genotype and up to 30% inhibition of morphine uptake, but only for the OCT1*2 allele. At high concentrations expected in the gastrointestinal tract, ranitidine showed strong inhibition (by more than 70%) with all substrates and genotypes tested (Fig 3).

### Ranitidine as a substrate of OCT2 and effects of the Ala270Ser polymorphism on OCT2-mediated uptake of ranitidine

**Fig 4:**
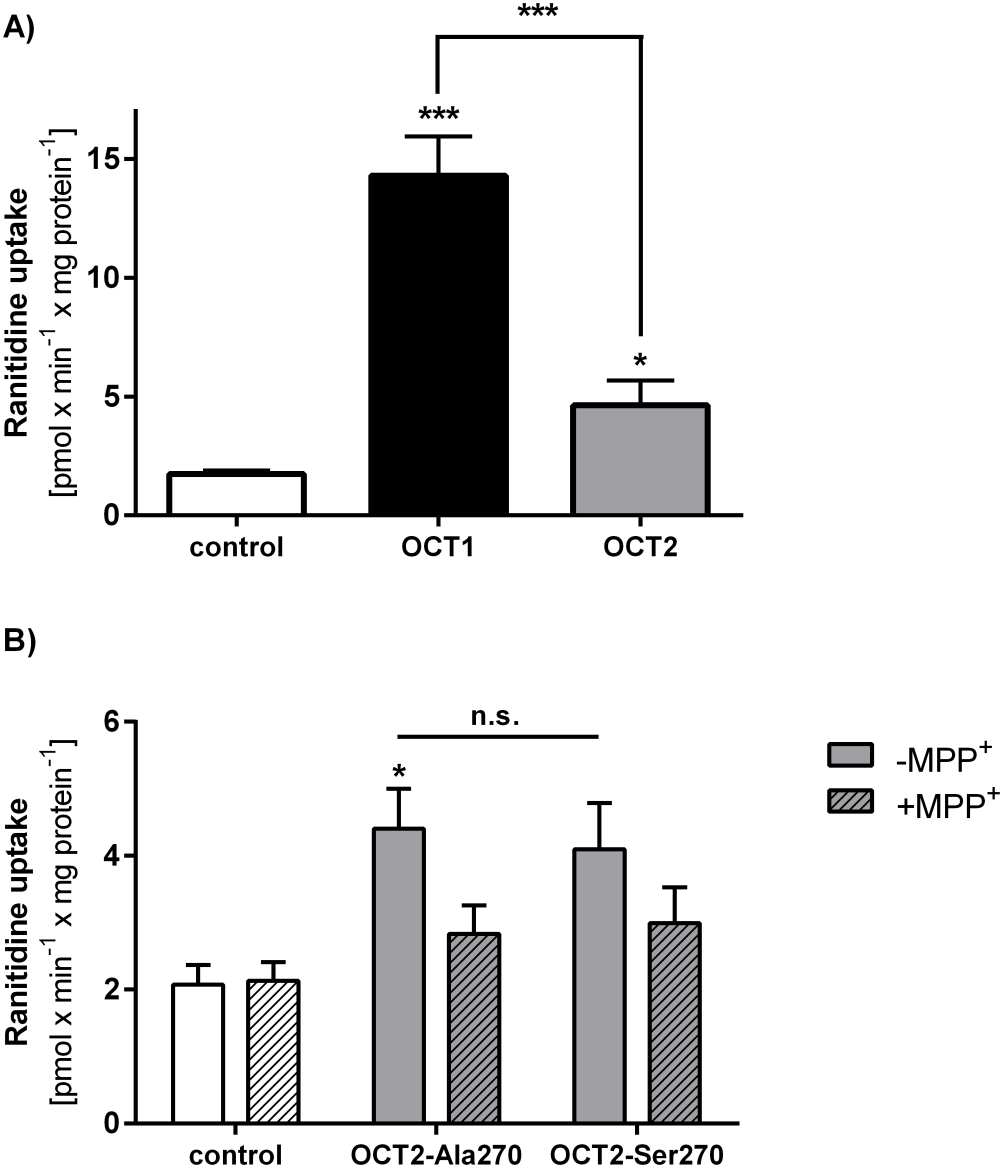
OCT2-mediated uptake of ranitidine and effects of the Ala270Ser polymorphism. A) Comparison between OCT1 and OCT2-mediated uptake of ranitidine. Cells overexpressing OCT1 or OCT2 and control cells were incubated for 2 min with 1 μM ranitidine. The data is shown as means and standard error of the means of at least three independent experiments. Significance was calculated using ANOVA with Tukey’s HSD post hoc comparisons to the control cells or between OCT1 and OCT2. * P<0.05, *** P<0.001. B) Effects of the Ala270Ser polymorphism on the OCT2-mediated uptake of ranitidine. HEK293 T-REx™ cells stably transfected to overexpress *OCT2* reference (Ala270) and variant (Ser270) alleles, and control cells transfected with the empty vector pcDNA5 were incubated with 1 μM ranitidine for 2 min in the presence or the absence of 1 mM MPP^+^. The data is shown as means and standard error of the means of three independent experiments performed in duplicates. Significance was calculated using ANOVA with Tukey’s HSD post hoc comparisons. * P<0.05.

We also analyzed whether OCT2, the major OCT isoform expressed in the human kidney, mediates the cellular uptake and thus may potentially contribute to the renal secretion of ranitidine. After incubation for 2 min with 1 μM ranitidine OCT2-overexpressing HEK293 cells showed a 3.3-fold higher ranitidine uptake than the control cells (Fig 4A). However, the uptake was significantly lower than the one observed in OCT1 under the same conditions (7.8-fold increase by the OCT1-overexpressing compared to the control cells). Due to the small difference in uptake between the OCT2-overexpressing and the control HEK293 cells at higher ranitidine concentrations, it was not possible to measure a saturable uptake and to calculate uptake kinetic parameters for OCT2. In comparison to the Ala270 allele, the Ser270 allele showed a limited reduction of ranitidine uptake by 9% which was not significant (Fig 4B). The uptake could be inhibited by MPP^+^, but there was no difference between the *OCT2* alleles.

## Discussion

In this study we confirmed that ranitidine is a substrate of the human hepatic uptake transporter OCT1 and demonstrated that genetic polymorphisms in *OCT1* lead to a significant reduction or even complete loss of ranitidine uptake. In the human liver, the uptake via OCT1 may thus be the most important step prior to metabolic degradation of ranitidine by flavin-containing monooxygenases (FMOs) and cytochrome P450 (CYP) enzymes and this uptake may be substantially reduced in genetically-determined poor OCT1 transporters.

The clinical impact of these findings can currently only be speculated on by analogy to similarly hydrophilic OCT1 substrates like sumatriptan. The affinity and capacity of OCT1 to transport ranitidine are similar to the affinity and capacity for sumatriptan transport. Sumatriptan pharmacokinetics in humans is strongly affected by *OCT1* polymorphisms [36]. Ranitidine hepatic clearance is estimated to be between 30 and 50% of the total systemic clearance [8, 9], but similarly to sumatriptan also the hepatic accumulation may be affected. The volume of distribution of ranitidine is close to 1.4 l/kg indicating deep compartment accumulation of the drug. Our data suggest also that due to the strong ability of OCT1 to transport ranitidine and due to the very strong OCT1 expression in the sinusoidal hepatocyte membrane an accumulation of ranitidine in the liver may be one explanation for the high volume of distribution. Indeed a recent study of Sundelin *et al*. elegantly demonstrated this for metformin [31]. Both the effects on the hepatic ranitidine clearance and volume of distribution will be strongly OCT1 dependent and should be observed in individuals with OCT1 deficiency.

Our data suggest that poor OCT1 transporters (homozygous or compound heterozygous carriers of the *OCT1* alleles *2, *3, *4, *5, or *6) will have strongly reduced or completely absent uptake of ranitidine in the liver. This represents 7% of the European and White American population [19].

The loss of transport activity by allelic variants OCT1*5 and *6 is caused by complete failure to localize in the plasma membrane [14, 19, 52]. Also the reduction of OCT1 activity by the allelic variant OCT1*3 is due to a reduced localization in the plasma membrane. A reduction of plasma membrane localization by more than 80% has been reported for OCT1*3 (R61C) [52]. This suggests that the v_max_ reduction that we observed with ranitidine (Fig 2E and Table 2) could be completely explained by the impaired plasma membrane localization. In contrast, no reduction of plasma membrane localization could be measured for OCT1*2 [52] and several independent studies have shown that OCT1*4 and *10 are correctly localized in the plasma membrane [19, 34, 52]. Therefore, these three polymorphisms may be expected to have direct effects on OCT1 turnover rates. Development of methods for precise quantification of the OCT1 molecules that are localized in the plasma membrane should help address this quantitatively. However, we should be aware of the limitations of the existing methods for plasma membrane purification [55].

Accounting for the high availability of ranitidine, about 1.5 million poor OCT1 transporters could be estimated to use ranitidine regularly. It could be expected that these individuals will have an up to 50% increase in the plasma concentration of ranitidine and may have a substantial reduction of the hepatic ranitidine concentration. As we learned from other drugs [36] genetically-determined loss of OCT1 activity may have comparable effects on pharmacokinetics as hepatic deficiency. Indeed, some alterations in ranitidine half-life, distribution, clearance, and bioavailability were reported for patients with hepatic dysfunction [5]. However, ranitidine has a broad therapeutic index. Even dosages exceeding the single dose by more than 20-fold were not associated with a higher prevalence of adverse reactions [56]. Furthermore, in long-term toxicity tests performed in animals the plasma concentrations of ranitidine exceeded the human maximal plasma concentration by 50-fold without significant adverse reactions [5]. Therefore, the expected limited increase in ranitidine plasma concentrations in poor OCT1 transporters by 50% or more is not expected to lead to a substantial increase in the risk of ranitidine-related systemic adverse reactions.

On the other hand, poor OCT1 transporters of ranitidine may have substantially lower ranitidine concentrations in hepatocytes and probably a reduced risk for hepatic toxicity. Indeed, ranitidine administration has been associated with an increase of the transaminases ALT and AST [5] and very rare cases of severe hepatotoxicity following ranitidine administration were reported [57]. The severe hepatotoxicity is currently not fully understood and thus termed idiosyncratic [7]. In rats ranitidine administration was shown to inhibit liver regeneration [58]. It will be interesting to analyze whether poor OCT1 transporters have a less pronounced increase in ALT and AST and whether they are at reduced risk of severe hepatotoxicity after ranitidine administration.

At clinically used doses ranitidine is apparently rather safe and only few ranitidine-related drug-drug-interactions have been described [6, 59, 60]. However, high-dose ranitidine may still be interesting as a probe drug to inhibit OCT1-mediated uptake of other drugs *in vivo*. To this end we should be able to estimate whether ranitidine may inhibit OCT1 at concentrations reached in humans and whether common genetic variants in *OCT1* may modulate this inhibition. Our data strongly suggest that ranitidine may reach concentrations sufficient to inhibit OCT1 uptake only in the intestine (Fig 3, Table 3). The expression and the exact localization of OCT1 in the intestine are still questionable. Although modern protein analytic methods suggest the presence of OCT1 in the small intestine [61], the OCT1 mRNA levels in the intestine are up to 800-fold lower than those in the liver [62, 63]. Furthermore, the exact subcellular localization of OCT1 in the intestine is controversial. Whereas Müller *et al*. showed OCT1 to be located on the basolateral membrane of enterocytes [53], Han *et al*. showed an apical localization of OCT1 [64]. Therefore, ranitidine inhibition may be useful to elucidate whether OCT1 plays a functional role in the intestine in humans. Furthermore, our data showed that ranitidine concentrations in the intestine are sufficient to inhibit OCT1-mediated uptake of all substrates tested, with only minor effects of genetic polymorphisms.

**Table 3:** Genotype and substrate-dependent variations in the potency of ranitidine to inhibit OCT1-mediated uptake related to the expected ranitidine concentration in the gastrointestinal tract [*I*]_2_ and in plasma [*I*]_1_.

Substrate	OCT1 allele	IC_50_ [μM]	[*I*]_2_ [μM]	[*I*]_2_/IC_50_	[*I*]_1_ [μM]	[*I*]_1_/IC_50_
Morphine	OCT1*1	45.5 ± 5.49	3800	**83.5**	2.2	0.048
	OCT1*2	19.5 ± 0.0		**194.9**		**0.113**
Metformin	OCT1*1	20.93 ± 3.2		**181.56**		**0.105**
	OCT1*2	14.87 ± 3.52		**255.55**		**0.148**
ASP^+^	OCT1*1	336.59 ± 3.92		**11.29**		0.007
	OCT1*2	155.21 ± 10.49		**24.48**		0.014
MPP^+^	OCT1*1	81.91 ± 5.52		**46.39**		0.027
	OCT1*2	34.43 ± 2.71		**110.37**		0.064

IC_50_ –half maximal inhibitory concentration, [*I*]_1_ –mean unbound ranitidine steady state total C_max_ at highest clinical dose (300 mg), [*I*]_2_ –theoretical maximal gastrointestinal ranitidine concentration after oral administration (highest clinical dose (300 mg) in a volume of 250 ml)

The ratios are highlighted in bold if exceeding the threshold of 0.1 for [*I*]_1_/IC_50_ and 10 for [*I*]_2_/IC_50_. The thresholds are regarded as indicative for potential drug-drug interactions where further *in vivo* studies are recommended [65].

The effects of ranitidine on the intestinal uptake of OCT1 may be more complex than simple OCT1 inhibition. Due to its therapeutic action, ranitidine may increase the intestinal pH [8], which then leads to improved absorption by passive diffusion of weakly basic drugs—the typical substrates of OCT1. This effect may compensate the OCT1 inhibitory effects by ranitidine in the intestine.

On the other hand, following the formal criteria for evaluating the potential for drug-drug interactions [65], potential drug-drug interactions at liver OCT1 would have required further attention. We observed ratios of plasma concentration to IC_50_ ([*I*]_1_/IC_50_) of greater than 0.1 (Table 3). This indicates a potential for drug-drug interactions and is suggested in the guidelines as a criterion for further *in vivo* analyses [65]. However, ranitidine has been broadly used for many years without strong drug-drug-interactions to be reported.

Regarding the genotype-specific effects on OCT1 inhibition with ranitidine, our data suggest that by co-administration of ranitidine carriers of OCT1*2 alleles will have lower uptake of morphine in the liver than carriers of the reference OCT1*1 allele. This may be especially relevant for one of 17 Europeans and White Americans who is a heterozygous carrier of an OCT1*2 and a completely deficient allele, or a homozygous OCT1*2 allele carrier. Interestingly, we observed an induction, instead of an inhibition, of OCT1-mediated morphine uptake at low concentrations (Fig 3). This may be relevant as these concentrations are close to the plasma concentrations of ranitidine in humans. This effect has been reported for other transporters before [66, 67] and points to positive interaction of the two substrates on the binding site—an interaction that seems to be missing in the OCT1*2 allele.

**Fig 5:**
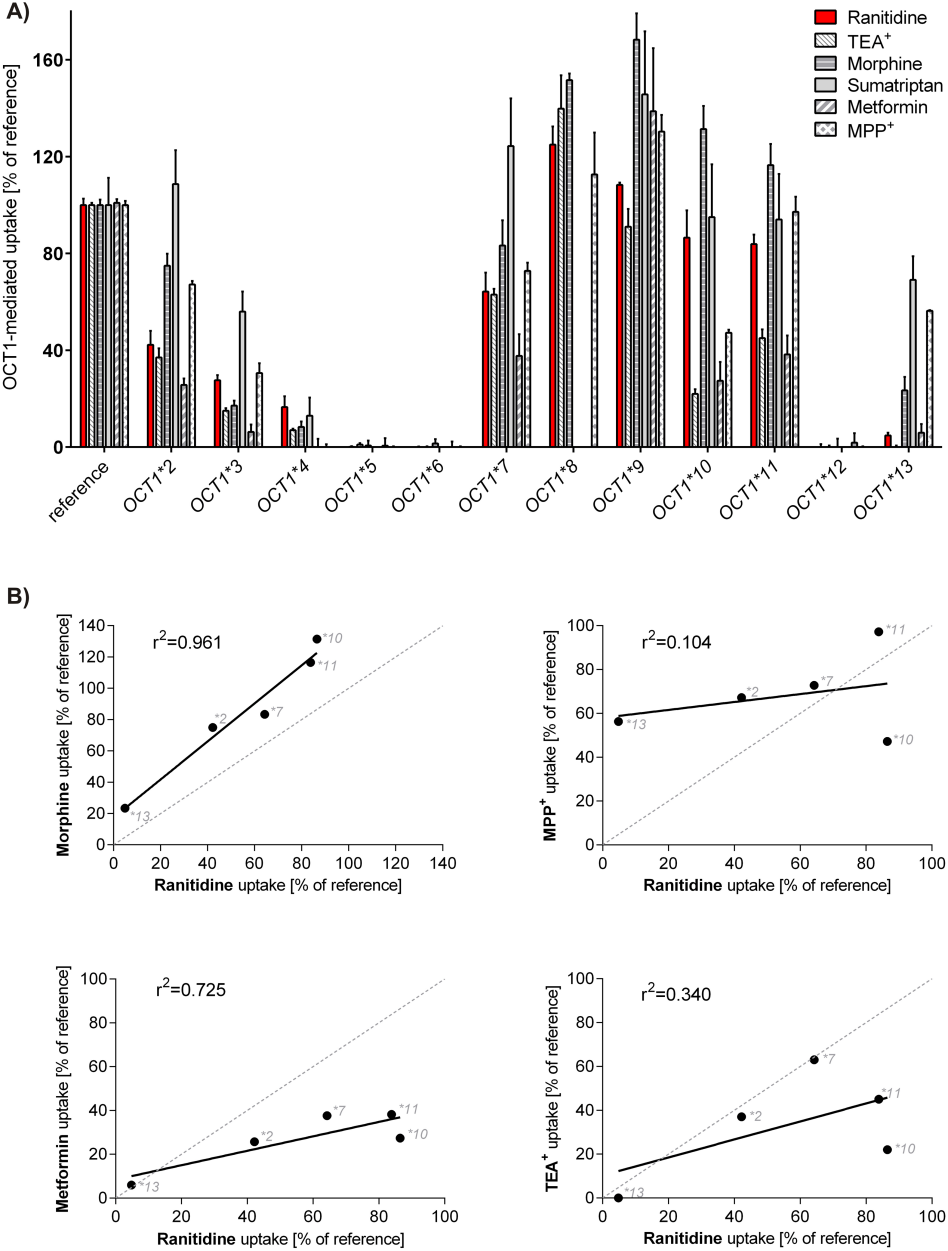
Comparison between the effects of genetic polymorphisms on the uptake of ranitidine and other OCT1 substrates. A) Comparative representation of the effects of *OCT1* allelic variants on the uptake of ranitidine, TEA^+^, morphine, sumatriptan, metformin, and MPP^+^. HEK293 T-REx^™^ cells overexpressing the reference *OCT1* allele and common OCT1 allelic variants were incubated for 2 min with 1 μM ranitidine, 5 μM TEA^+^, 1 μM morphine, 0.1 μM sumatriptan, 5 μM metformin, or 10 μM MPP^+^. The OCT1-mediated uptake was calculated by subtracting the uptake of control cells from the uptake of OCT1-overexpressing cells. The data is shown as means and standard error of the means of at least three independent experiments. B) Correlation of the uptake rates of ranitidine with the model substrates MPP^+^ and TEA^+^ and the clinically relevant substrates metformin and morphine. Analyzed were all *OCT1* alleles that have correct localization in the cell membrane [19] and show a substrate-specific loss of activity, i.e. OCT1*2, OCT1*7, OCT1*10, OCT1*11, and OCT1*13. The uptake rates are presented as percentage of the reference *OCT1* allele. Solid lines represent linear regression; dashed lines represent an optimal theoretical correlation with identical effects of the allelic variants on both substrates. The values for the uptake of morphine, metformin, sumatriptan, TEA^+^, and MPP^+^ are obtained from our previously published studies [12, 19, 36].

Beyond potential relevance for drug pharmacokinetics in humans our data may help to gain some insight into the mechanisms leading to the polyspecificity of the OCT1 transporter. It is supposed that OCT1 has multiple, possibly overlapping substrate binding sites [68, 69]. Even though several amino acids have been identified to be important for OCT1 substrate translocation, the exact binding sites remain unknown, as the 3D structure of OCT1 has not yet been determined. Comparing the effects of *OCT1* allelic variants on the uptake of different substrates may help to reveal substrate-specific interactions with the transporter. Such comparisons have been performed to show similarities between thiamine and metformin [15] and between other substrates before [19]. The effects of common *OCT1* genetic variants on ranitidine uptake correlated very well with the effects on morphine uptake (r^2^ = 0.961). Weaker correlation was observed with metformin and only limited correlations with MPP^+^ or TEA^+^ uptake (Fig 5). This suggests that ranitidine and morphine share highly overlapping binding sites. Furthermore, the effects of *OCT1* allelic variants on TEA^+^ and metformin uptake correlated strongly with each other, but none of them correlated with ranitidine uptake. Interestingly, *OCT1* alleles OCT1*10 and OCT1*11 did not affect ranitidine uptake, but strongly reduced TEA^+^ and metformin uptake (Fig 5B). When excluding these two alleles from the correlation, ranitidine uptake highly correlates with both TEA^+^ and metformin uptakes. This indicates that serine_189_ (Ser189Leu is characteristic for OCT1*10) and isoleucine_449_ (Ile449Thr is characteristic for OCT1*11) are involved in the transport of TEA^+^ and metformin, but do not play a substantial role in the transport of ranitidine.

Another major finding in our study is that ranitidine is only marginally transported by human OCT2. Renal excretion is the major route of ranitidine elimination in humans [8]. The renal clearance of ranitidine is above 400 ml/min [8], suggesting that tubular secretion plays the predominant role in ranitidine renal elimination. A study of van Crugten *et al*. showed a reduction of renal clearance of ranitidine by over 40% by cimetidine, suggesting a substantial involvement of organic cation transporters [70], and the basolaterally expressed organic cation transporter OCT2 was suggested to play a role in renal ranitidine elimination. However, we observed only a limited uptake of ranitidine via OCT2 compared to OCT1. The limited ability of OCT2 to transport ranitidine we observed here is in concordance with previously published data [11]. Furthermore, the OCT2-mediated uptake of ranitidine was not substantially affected by the Ala270Ser substitution, the only common genetic polymorphism suggested to affect OCT2 function [47]. In line with this, genetic polymorphisms in MATE1, but not in OCT2 were found to affect metformin pharmacokinetics under ranitidine administration [71]. Therefore it could be concluded that OCT2 and common genetic polymorphisms in particular may play only a limited role in the renal elimination of ranitidine.

Considering that OCT2 is the only basolaterally expressed organic cation transporter in the human proximal tubules, it remains unclear how the strong tubular secretion may be mediated. Interestingly, also organic anion transporters from the SLC22 family that are basolaterally expressed in the human kidney—OAT1, OAT2, and OAT3—were shown to transport ranitidine *in vitro* [72]. Furthermore, co-administration of the OAT-inhibitor probenecid in dogs reduced ranitidine renal clearances by two-fold and basolateral uptake by four-fold [73]. It will be interesting to evaluate experimentally the role of these transporters in the renal elimination of ranitidine in humans.

The major limitation of our study is that it contains only *in vitro* analyses. Regarding the effects of OCT1 polymorphisms our data suggest that there should not be substantial effects on ranitidine pharmacokinetics and no further *in vivo* studies are needed. In the case of drug-drug interactions, especially regarding an intestinal absorption of ranitidine, further studies in humans may be indicated.

In conclusion, we demonstrated that ranitidine is a substrate of OCT1 and that common genetic polymorphisms in *OCT1* lead to a substantial reduction or even complete abolishment of OCT1-mediated ranitidine uptake. Due to reduced hepatic uptake poor OCT1 transporters are expected to have increased ranitidine plasma concentrations by 50% or more. However, as ranitidine has a very broad therapeutic range, this increase is not expected to have clinical consequences. Our data also suggest that ranitidine may be utilized as an *in vivo* inhibitor to elucidate the role of OCT1 in the human gut. Last but not least, effects of *OCT1* polymorphisms on ranitidine uptake correlated very strongly with effects on morphine uptake, which suggests highly overlapping binding sites of these two substrates in OCT1. These binding sites partially differ from the binding sites of metformin or the model substrate MPP^+^.

## Supporting information

S1 Fig Effects of *OCT1* allelic variants on ranitidine uptake. HEK293 T-REx^™^ cells overexpressing the reference allele (OCT1*1B) and common *OCT1* allelic variants (the allelic variants are listed in Fig 2A) were incubated with 10 μM ranitidine for 2 min. The OCT1-mediated uptake was calculated by subtracting the uptake of control cells from the uptake of OCT1-overexpressing cells. The data is shown as means and standard error of the means of at least three independent experiments.
(TIF)
